# Molecular Identification of Chronic Bee Paralysis Virus Infection in *Apis mellifera* Colonies in Japan

**DOI:** 10.3390/v4071093

**Published:** 2012-06-29

**Authors:** Tomomi Morimoto, Yuriko Kojima, Mikio Yoshiyama, Kiyoshi Kimura, Bu Yang, Tatsuhiko Kadowaki

**Affiliations:** 1 Graduate School of Bioagricultural Sciences, Nagoya University, Chikusa, Nagoya 464-8601, Japan; Email: morimoto-tomomi@api3838.co.jp (T.M.); atmkun0517@yahoo.co.jp (Y.K.); 2 Honeybee Research Unit, Animal Breeding Research Group, Animal and Reproduction Division, National Institute of Livestock and Grassland Science, 2 Ikenodai, Tsukuba, Ibaraki 305-0901, Japan; Email: yoshiyam@affrc.go.jp (M.Y.); kimura@affrc.go.jp (K.K.); 3 Department of Biological Sciences, Xi’an Jiaotong-Liverpool University, 111 Ren’ai Road, Suzhou Dushu Lake Higher Education Town, Jiangsu Province 215123, China; Email: Bu.Yang08@student.xjtlu.edu.cn

**Keywords:** chronic bee paralysis virus, *Apis mellifera*, *Apis cerana japonica*, phylogeny

## Abstract

Chronic bee paralysis virus (CBPV) infection causes chronic paralysis and loss of workers in honey bee colonies around the world. Although CBPV shows a worldwide distribution, it had not been molecularly detected in Japan. Our investigation of *Apis mellifera* and *Apis cerana japonica* colonies with RT-PCR has revealed CBPV infection in *A. mellifera* but not *A. c. japonica* colonies in Japan. The prevalence of CBPV is low compared with that of other viruses: deformed wing virus (DWV), black queen cell virus (BQCV), Israel acute paralysis virus (IAPV), and sac brood virus (SBV), previously reported in Japan. Because of its low prevalence (5.6%) in *A. mellifera* colonies, the incidence of colony losses by CBPV infection must be sporadic in Japan. The presence of the (−) strand RNA in dying workers suggests that CBPV infection and replication may contribute to their symptoms. Phylogenetic analysis demonstrates a geographic separation of Japanese isolates from European, Uruguayan, and mainland US isolates. The lack of major exchange of honey bees between Europe/mainland US and Japan for the recent 26 years (1985–2010) may have resulted in the geographic separation of Japanese CBPV isolates.

## 1. Introduction

Chronic bee paralysis virus (CBPV) causes chronic paralysis, an infectious disease of adult honey bees. The symptoms of this disease include severe trembling of the wings and bodies, and diseased bees often crawl on the ground. Some individuals become hairless and, thus, darker in appearance. Diseased bees die within a few days [1,2]. CBPV can persist as an unapparent infection but may multiply to high levels in honey bee colonies [3], causing significant losses [4]. Although CBPV shares several characteristics with the *Nodaviridae* and *Tombusviridae* virus families, CBPV is considered a new family of positive single-stranded RNA viruses. The sizes of the two major CBPV RNAs were determined to be 3674 bases for RNA 1 and 2305 bases for RNA 2 [5].

We previously conducted the first nationwide epidemiological survey in Japan of honey bee parasites and pathogens, to measure their impact on colonies of the non-native European honey bee *Apis mellifera* and of the native Japanese honey bee *Apis cerana japonica* [6]. We found that microsporidia, *Nosema ceranae*, but not *Nosema apis*, commonly infects *A. mellifera* colonies (64% of colonies tested). Honey bee viruses, such as deformed wing virus (DWV), black queen cell virus (BQCV), and Israel acute paralysis virus (IAPV), are also prevalent (>66% of colonies tested) in *A. mellifera* colonies. The prevalence of *Nosema* and viruses in *A. c. japonica* colonies is lower than that in *A. mellifera* colonies. Our survey also revealed a tracheal mite (*Acarapis woodi*) infestation in both honey bee species for the first time in Japan. These results demonstrate the infestation of native honey bees by parasite and pathogens of non-native honey bees that are traded globally [6]. Although CBPV shows a worldwide distribution [7], our previous study of seven viruses (ABPV, BQCV, CBPV, DWV, IAPV, KBV, and SBV) infecting healthy honey bee (*A. mellifera* and *A. c. japonica*) colonies in Japan did not detect CBPV in any of the samples analyzed. However, we later found CBPV by RNAseq analysis (high-throughput sequencing of mRNA) in dying workers from two colonies (Japan-2 and -5) used for pollination of strawberries in greenhouses. This observation led us to test for CBPV infection in both healthy and collapsing *A. mellifera* and *A. c. japonica* colonies.

## 2. Results

**Figure 1:**
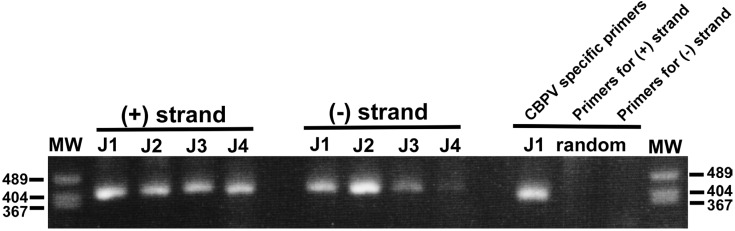
Molecular detection of the (+) and (−) strands of CBPV RNA2 in dying workers.(+) and (−) strands of CBPV RNA2 in dying workers from the *A. mellifera* Japan-1, -2, -3, and -4 colonies (J1 to J4) have been detected separately by strand-specific RT-PCR. Both (+) and (−) strand RNAs are present in the dying workers; however, the relative level of (−) strand RNA in those from the Japan-4 (J4) colony is low. Control PCR reactions were performed using CBPV cDNAs (Japan-1, J1) generated with random hexamer primers (“random”) and the CBPV-specific primers or using the two sets of primers used for detecting the (+) and (−) strands of CBPV RNA2. The sizes of three bands of the molecular weight marker (MW) are shown on both sides of the gel.

Healthy 60 *A. mellifera* and 55 *A. c. japonica* colonies without significant loss of workers were analyzed for CBPV infection by collecting and pooling 50 workers from a single colony. In addition, approximately 50 dying workers (crawling on the ground in front of the hive entrance) from a single colony were sampled and pooled for 29 collapsing *A. mellifera* colonies with significant loss of workers. This investigation has identified 3 more CBPV-infected *A. mellifera* colonies (Japan-1, -3, and -4) among the collapsing colonies. CBPV-positive *A. c. japonica* colonies have not been found. Table 1 summarizes the presence of pathogens and parasites in five CBPV-infected *A. mellifera* colonies with severe loss of workers. CBPV was not found in healthy colonies. Infection by multiple viruses was detected in four colonies (Japan-1, -2, -3, and -4), and *N. ceranae* is present in all colonies. Furthermore, the Japan-3 colony is also infested with tracheal mites. To examine a possible role of CBPV infection in the severe loss of workers, the level of viral replication was analyzed by detecting the (−) strand RNA of the viral genome. The (−) strand viral RNA is synthesized by RNA-dependent RNA polymerase (RdRP) using the (+) strand viral RNA genome as a template. This is then used as a template to synthesize the (+) strand viral RNA genome by RdRP. A similar level of both (+) and (−) strand RNAs were found in the dying workers from the Japan-1, -2, and -3 colonies, suggesting that CBPV proliferates in the workers themselves (Figure 1). To test for the specificity of detecting the (+) and (−) strands of CBPV RNA2 by strand-specific RT-PCR, we performed an additional control experiment, in which we analyzed CBPV cDNAs obtained with random hexamer primers. Although the CBPV-specific primers generated a positive band, the two sets of primers used for the strand-specific RT-PCR failed to give positive signals (Figure 1). The relative level of (−) strand RNA was low in dying workers from the Japan-4 colony, and neither (+) nor (−) strand RNAs were detected in dying workers from the Japan-5 colony with this method (data not shown). Thus, CBPV infection and proliferation may contribute to the loss of workers from the Japan-1, -2, and -3 but not from the Japan-4 and -5 colonies.

**Table 1:** Summary of pathogens and parasite present in CBPV-infected *A. mellifera* colonies.

Sample	Virus	Microsporidia	Protist	Tracheal mite	Severe loss of workers	Use for pollination
Japan-1	CBPV, BQCV, DWV	*N. ceranae*	−	−	+	−
Japan-2	CBPV, BQCV, DWV	*N. ceranae*	−	−	+	+
Japan-3	CBPV, BQCV, DWV, IAPV, SBV	*N. ceranae*	−	+	+	−
Japan-4	CBPV, BQCV, DWV, IAPV	*N. ceranae*	−	−	+	−
Japan-5	CBPV	*N. ceranae*	−	−	+	+

The presence of microsporidia (*N. apis* and *N. ceranae*), protists (*Crithidia mellificae* and *Apicystis bombi*), and tracheal mite in five CBPV-infected *A. mellifera* colonies (Japan-1 to -5) was examined by PCR. A severe loss of workers was found with all colonies, and Japan-2 and -5 colonies were used for pollination in strawberry greenhouses.

**Figure 2:**
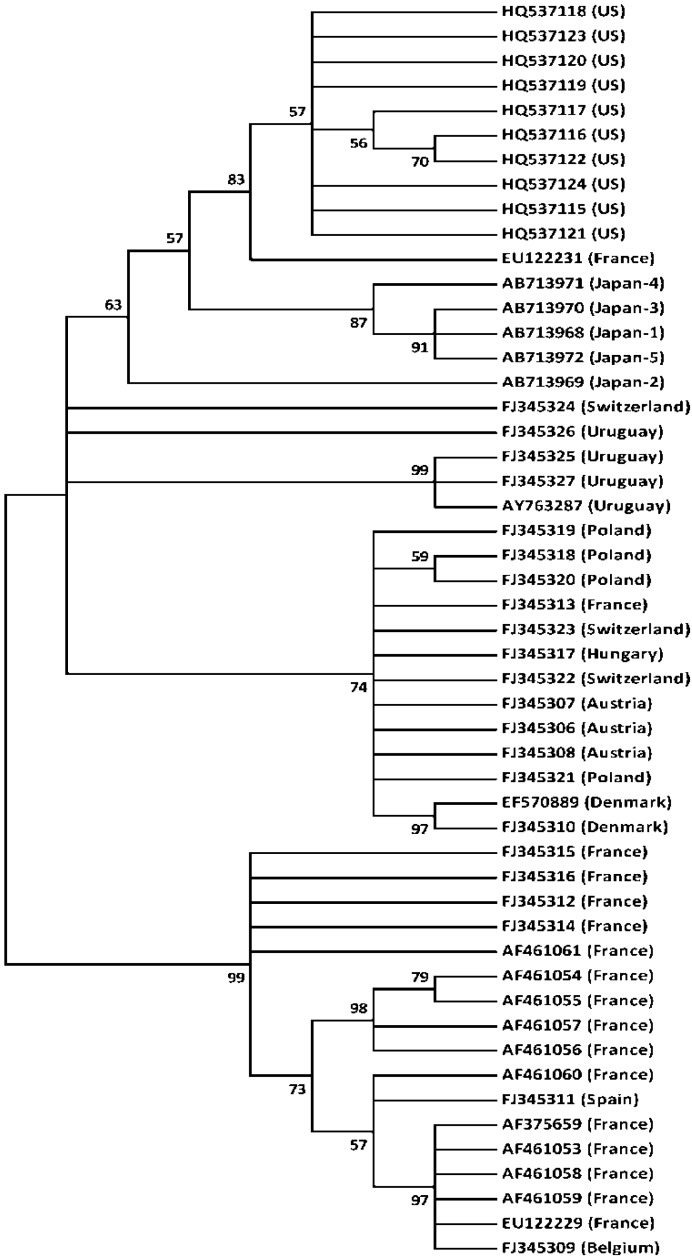
Phylogeny of Japanese, European, Uruguayan, and mainland US CBPV isolates. The condensed phylogenetic tree (bootstrap values <50%) based on alignment of the putative RdRP gene sequences of Japanese, European, Uruguayan, and mainland US CBPV isolates was constructed using the maximum likelihood method. The number of each node represents the bootstrap value resulting from 1000replicates. Each isolate is indicated by its accession number.

The phylogeny of Japanese, European, Uruguayan, and mainland US CBPV isolates was constructed using the maximum likelihood method and the putative RdRP coding sequences (Figure 2). This gene was selected for the analysis since many sequences of the isolates from different countries are available in a GenBank. As previously reported [8,9], the European isolates are grouped into two major clades, except two isolates: one French (EU122231) and one Swiss (FJ345324). Uruguayan isolates also cluster with one exception (FJ345326). Ten mainland US isolates form the independent clade, and four Japanese isolates (Japan-1, -3, -4, and -5) also independently cluster. However, one Japanese isolate (Japan-2) is basal to the mainland US and Japanese isolates (Figure 2). Although there are several exceptions, European, mainland US, Uruguayan, and Japanese isolates show the trends of geographic separation.

## 3. Discussion

This reports a molecular identification of CBPV in Japan, and is consistent with its worldwide distribution [7], which may result from global trade of *A. mellifera* queens among many countries. Although our current investigation has revealed a lower prevalence of CBPV in Japan, relative to other viruses, including DWV, BQCV, IAPV, and SBV [6], this prevalence could be underestimated because one primer set we used for primary screening may fail to detect more divergent strains of CBPV infecting both *A. mellifera* and *A. c. japonica*. We have not found CBPV-positive *A. c. japonica* colonies in the current study, suggesting that CBPV may have a species barrier for infection, as demonstrated with SBV [6,10]. Alternatively, the low prevalence in *A. mellifera* colonies may, itself, explain the absence of CBPV in *A. c. japonica* colonies. A survey of honey bee viruses in China indicated that the prevalence of CBPV was 11.8% in 34 apiaries with *A. mellifera* colonies [11], and appears to be higher than that in Japan (5.6%). CBPV infection is not thought to induce massive colony losses, but it could contribute to a severe loss of workers in an infected colony, as we observed with the Japan-1, -2, and -3 colonies. However, these colonies were also infected with other viruses and *N. ceranae* (Table 1), suggesting that CBPV may not be the single cause for the loss of workers. The relative level of (−) strand RNA was high in dying workers from the Japan-2 colony used for pollination in a strawberry green house. It demonstrates that CBPV actively proliferates in these workers (Figure 1), and this is likely to result from the immune suppression of honey bees used for long-term pollination in greenhouses [12].

The geographic separation of Japanese CBPV isolates from those of other countries is quite similar to the separation we previously observed with DWV [6]. The lack of major honey bee trade between Europe/mainland US and Japan in the last 26 years (1985–2010) may have resulted in the geographic separation of Japanese isolates. Twenty-seven and 100 queen bees were imported from Italy and Switzerland to Japan in 1987 and 1997, respectively. Two hundred and five queen bees were imported from Yugoslavia during 1989–1991, and 2138 queen bees were imported from Slovenia during 1998–2010. Similarly, 77 queen bees were imported from mainland US during 1985–1992. This phylogenetic relationship did not change even when the tree was rooted by including an outgroup virus, for example, BQCV (data not shown). We also constructed a phylogenetic tree based on a 527-nucleotide sequence encoding the putative structural proteins of CBPV isolates from Japan and other countries. The putative structural protein gene was previously identified in RNA2 [5]. The Japanese isolates also cluster, but form an independent clade along with two Uruguayan isolates and one Swiss isolate (data not shown). However, as described by [8], the amino acid sequences of these structural proteins show a very low degree of divergence, suggesting that they may not be suitable for inferring the phylogeny. Since Japan has imported most of its honey bee queens from Hawaii (12486 queen bees), New Zealand (51761 queen bees), and Australia (87698 queen bees) in the last 26 years, it would be interesting to determine the phylogeny of CBPV isolates from Japan and from these areas. Such a phylogeny may reveal the role of honey bee trade for viral transmission between countries.

## 4. Experimental Section

### 4.1. Honey Bee Sample Collection

*A. mellifera* colonies were sampled from 60 apiaries in 44 prefectures in Japan in collaboration with the Japan Beekeeping Association. Fifty workers were collected and pooled from a single colony in each apiary. A total of 55 *A. c. japonica* samples (each sample: 50 pooled workers from a single colony) were collected from 30 managed and 25 feral colonies. All of the above were healthy colonies and the workers were collected from brood nests inside the hives for managed colonies and at the hive entrances for feral colonies, respectively. In addition, approximately 50 dying workers (crawling on the ground in front of the hive entrance) were sampled from 29 collapsing *A. mellifera* colonies.

### 4.2. RT-PCR Detection of Viruses

Total RNA was isolated from 20 workers collected from single colonies as above by using Trizol reagent (Invitrogen). Total RNA (1 µg) was used for reverse transcription reaction by using ReverTra Ace reverse transcriptase (TOYOBO) and a random primer. The reverse transcription products were then used for PCR with KOD FX DNA polymerase (TOYOBO) and the primer set for detecting CBPV (5´-GACCCCCGTTGGAACGACGC-3´ and 5´-CGGACGACGATTGGCGCTCA-3´). These primers were newly designed according to the RNA2 sequence we identified in dying workers from two colonies (Japan-2 and -5) used for pollination of strawberries in greenhouses by RNAseq analysis. The expected size of the amplified product is 233 bp (bases 131–363) [5] which is clearly visible by 2% agarose gel electrophoresis. Honey bee *EF-1alpha* was used as a positive control to verify the quality of RNA extraction as well as reverse transcription, and also serves as an internal loading standard. The primer set, 5´-TGCAAGAGGCTGTTCCTGGTGA-3´ and 5´-CGAAACGCCCCAAAGGCGGA-3´, was used. The thermal cycling conditions were as follows: one cycle of initial denaturation at 94 °C for 2 min, 35 cycles of denaturation at 98 °C for 10 s, annealing at 55 °C for 30 s, and extension at 68 °C for 30 s. The concentrations of each primer and dNTPs for PCR were 0.3 µM and 0.4 mM, respectively. A negative control lacking template DNA and a positive DNA control were performed for each PCR reaction. Positive identification was confirmed by sequencing the PCR products. In five CBPV-positive *A. mellifera* colonies (Japan-1 to -5 in Table 1), the infection of ABPV (acute bee paralysis virus), BQCV, CBPV, DWV, IAPV, KBV (Kashmir bee virus), and SBV was also tested as previously described in [6].

To specifically detect the (+) and (−) strands of CBPV RNA2 (bases 367–765) [5], a reverse transcription reaction was carried out using the following primers: 5´-AGCCTGCGCACCGTGGGCGATCCTCGATGGTGCGGAG-3´ for (+) strand RNA detection and 5´-AGCCTGCGCACCGTGGCGACCACGCCGGTCCATTCT-3´ for (−) strand RNA detection. The reverse transcription products were then column purified to remove above primers, and then 25-fold dilutions were made with H_2_O prior to PCR. A tag primer (5´-AGCCTGCGCACCGTGG-3´) and 5´-CGACCACGCCGGTCCATTCT-3´ and the tag primer and 5´ GCGATCCTCGATGGTGCGGAG 3´ were used for PCR to detect the (+) and (−) strands of CBPV RNA2, respectively. The thermal cycling conditions with the annealing temperature at 55 °C were the same as above. For a control experiment of the strand-specific RT-PCR, CBPV cDNAs were synthesized with total RNA isolated from honey bees of Japan-1 colony (Table 1) and random hexamer primers. PCR was then carried out with the CBPV-specific primers and the two sets of primers used for the strand-specific RT-PCR. The PCR product was analyzed by 2% agarose gel electrophoresis.

### 4.3. PCR Detection of N. ceranae and N. apis

Total genomic DNA was isolated with 20 dying workers from each of five CBPV-infected *A. mellifera* colonies (Japan-1 to -5 in Table 1) using DNAzol reagent (Invitrogen), and dissolved in 100 µL of 8 mM NaOH followed by neutralization by adding 1 µL of 1 M HEPES. Total DNA (0.1 µg) was used for PCR with KOD FX DNA polymerase and the following primer sets: 5´-CCATTGCCGGATAAGAGAGT-3´ and 5´-CCACCAAAAACTCCCAAGAG-3´ for *N. apis*, and 5´-CGGATAAAAGAGTCCGTTACC-3´ and 5´-TGAGCAGGGTTCTAGGGAT-3´ for *N. ceranae* [13]. As a control, a honey bee genomic DNA fragment encoding a part of AmHsTRPA [14] was PCR amplified with the following primers: 5´-CACGACATTCAAGGTTTAAGAAATCACG-3´ and 5´-TCAGTTATTCTTTTCCTTTGCCAGATTT-3´. The thermal cycling conditions with the annealing temperature at 55 °C and the gel electrophoresis were the same as above. A negative control lacking template DNA and a positive DNA control were performed for each PCR reaction. Positive identification was confirmed by sequencing the PCR products.

### 4.4. PCR Detection of Tracheal Mite (A. woodi)

Total genomic DNA prepared above was used for PCR detection of tracheal mite with the primer set (5´-TCTTCAATTTTAATTATACGT-3´ and 5´-CAAAAATCAGAATAAATGTTGAAATA-3´) as described in [15]. The thermal cycling conditions with the annealing temperature at 55 °C and the gel electrophoresis were the same as above. A negative control lacking template DNA and a positive DNA control were performed for each PCR reaction. Positive identification was confirmed by sequencing the PCR products.

### 4.5. PCR Detection of Crithidia mellificae and Apicystis bombi

Total genomic DNA prepared above was used for PCR detection of *C. mellificae* and *A. bombi* as described in [16]. The following primer sets: 5´-CTTTTGGTCGGTGGAGTGAT-3´ and 5´-GGACGTAATCGGCACAGTTT-3´ for *C. mellificae* and 5´-CCAGCATGGAATAACATGTAAGG-3´ and 5´-GACAGCTTCCAATCTCTAGTCG-3´ for *A. bombi* were used. The thermal cycling conditions with the annealing temperature at 55 °C and the gel electrophoresis were the same as above. A negative control lacking template DNA and a positive DNA control were performed for each PCR reaction. Positive identification was confirmed by sequencing the PCR products.

### 4.6. Construction of Phylogenetic Tree of CBPV

To construct phylogenetic tree of CBPV isolates from *A. mellifera*, PCR was first carried out, and then the amplified bands were sequenced. A primer set (5´-GATGTGATAAGWTSHSMTGGYWAACASRRT-3´ and 5´-ATGTGGCTTGCGACAACTCAGAAACAACTC-3´) was used for the first PCR, and then the following primer set: 5´-TAYGAGYGATTTYTTGRGATCGAYTTCGCT-3´ and 5´-TGTAYTCGRCCTGATTRACGACRTTAGC-3´ was used for the second PCR to amplify the 391-nucleotide sequence (bases 958–1348) [5] encoding the putative RdRP gene in five Japanese isolates. This gene was previously identified in RNA1 [5]. All determined sequences were deposited into the DDBJ database (Figure 1). To reveal the phylogenetic relationship between Japanese CBPV and isolates from other countries, all sequences were first aligned using the MUSCLE program [17], and then the Tamura-Nei distance substitution with a discrete gamma distribution model (TN93+G) was selected as the best-fit substitution model. The condensed phylogenetic tree was then constructed using the maximum likelihood method and a bootstrap value of 1000 replicates with MEGA5 [18].

## 5. Conclusions

Our investigation of *A. mellifera* and *A. c. japonica* colonies with RT-PCR has revealed CBPV infection in *A. mellifera* but not *A. c. japonica* colonies in Japan. The prevalence of CBPV is low compared with that of other viruses: DWV, BQCV, IAPV, and SBV, previously reported in Japan. Because of its low prevalence (5.6%) in *A. mellifera* colonies, the incidence of colony losses by CBPV infection must be sporadic in Japan. Phylogenetic analysis demonstrates a geographic separation of Japanese isolates from European, Uruguayan, and mainland US isolates. The lack of a major exchange of honey bees between Europe/mainland US and Japan for the recent 26 years may have resulted in the geographic separation of Japanese CBPV isolates.

## References

[1] Bailey L., Ball B.V., Perry J.N. (1983). Honeybee paralysis: Its natural spread and its diminished incidence in England and Wales. J. Apic. Res..

[2] Ball B.V., Bailey L., Morse R.A., Flottum K. (1997). Viruses. Honey Bee Pests, Predators, & Diseases.

[3] Blanchard P., Ribière M., Celle O., Lallemand P., Schurr F., Olivier V., Iscache A.L., Faucon J.P. (2007). Evaluation of a real-time two-step RT-PCR assay for quantification of Chronic bee paralysis virus (CBPV) genome in experimentally-infected bee tissues and in life stages of a symptomatic colony. J. Virol. Methods.

[4] Allen M., Ball B.V. (1996). The incidence and world distribution of honey bee viruses. Bee World.

[5] Olivier V., Blanchard P., Chaouch S., Lallemand P., Schurr F., Celle O., Dubois E., Tordo N., Thiéry R., Houlgatte R. (2008). Molecular characterization and phylogenetic analysis of Chronic bee paralysis virus: A honey bee virus. Virus Res..

[6] Kojima Y., Toki T., Morimoto T., Yoshiyama M., Kimur A.K., Kadowaki T. (2011). Infestation of Japanese native honey bees by tracheal mite and virus from non-native European honey bees in Japan. Microbial. Ecol..

[7] Ribière M., Olivier V., Blanchard P. (2010). Chronic bee paralysis: A disease and a virus like no other?. J. Invertebr. Pathol..

[8] Blanchard P., Schurr F., Olivier V., Celle O., Antùnez K., Bakonyi T., Berthoud H., Haubruge E., Higes M., Kasprzak S. (2009). Phylogenetic analysis of the RNA-dependant RNA polymerase (RdRp) and a predicted structural protein (pSP) of the chronic bee paralysis virus (CBPV) isolated from various geographical regions. Virus Res..

[9] Chen Y.P., Evans J.D., Pettis J.S. (2011). The presence of chronic bee paralysis virus infection in honey bees (*Apis mellifera* L.) in the USA. J. Apic. Res..

[10] Grabensteiner E., Ritter W., Carter M.J., Davison S., Pechhacker H., Kolodziejek J., Boecking O., Derakhshifar I., Moosbeckhofer R., Licek E. (2001). Sacbrood virus of the honeybee (*Apis mellifera*): Rapid identification and phylogenetic analysis using reverse transcription-PCR. Clin. Diagn. Lab. Immunol..

[11] Ai H., Yan X., Han R. (2012). Occurrence and prevalence of seven bee viruses in *Apis mellifera* and *Apis cerana* apiaries in China. J. Invertebr. Pathol..

[12] Morimoto T., Kojima Y., Toki T., Komeda Y., Yoshiyama M., Kimura K., Nirasawa K., Kadowaki T. (2011). The habitat disruption induces immune-suppression and oxidative stress in honey bees. Ecol. Evol..

[13] Chen Y.P., Evans J.D., Zhou L., Boncristiani H., Kimura K., Xiao T., Litkowski A.M., Pettis J.S. (2009). Asymmetrical coexistence of *Nosema ceranae* and *Nosema apis* in honey bees. J. Invertebr. Pathol..

[14] Kohno K., Sokabe T., Tominaga M., Kadowaki T. (2010). Honey bee thermal/chemical sensor, AmHsTRPA, reveals neofunctionalization and loss of transient receptor potential channel genes. J. Neurosci..

[15] Kojima Y., Yoshiyama M., Kimura K., Kadowaki T. (2011). PCR-based detection of a tracheal mite of the honey bee *Acarapis woodi*. J. Invertebr. Pathol..

[16] Meeus I., de Graaf D.C., Jans K., Smagghe G. (2010). Multiplex PCR detection of slowly-evolving trypanosomatids and neogregarines in bumblebees using broad-range primers. J. Appl. Microbiol..

[17] Edgar R.C. (2004). MUSCLE: Multiple sequence alignment with high accuracy and high throughput. Nucleic Acids Res..

[18] Tamura K., Peterson D., Peterson N., Stecher G., Nei M., Kumar S. (2011). EGA5: Molecular evolutionary genetics analysis using maximum likelihood, evolutionary distance; and maximum parsimony methods. Mol. Biol. Evol..

